# Normal Labor Accompanied with Abnormal Phenomena in Mother and Child

**Published:** 1886-07-20

**Authors:** J. McF. Gaston

**Affiliations:** Professor of the Principles and Practice of Surgery in the Southern Medical College, Atlanta, Georgia


### Southern Medical Record.

A MONTHLY JOURNAL OF PRACTICAL MEDICINE.

☞All Communications must be addressed to R. C. WORD, M. D., Managing Editor.

"*Quicquid Præcipies Esto Brevis.*"

VOL. XVI. ATLANTA, GA., JULY 20, 1886. No. 7

### ORIGINAL AND SELECTED ARTICLES.

### NORMAL LABOR ACCOMPANIED WITH ABNORMAL PHENOMENA IN MOTHER AND CHILD.

BY J. McF. GASTON, M. D.,

**Professor of the Principles and Practice of Surgery in the Southern Medical College, Atlanta, Georgia.**

I was called at half-past 10 o'clock a. m., on June 13, 1886, to Mrs. D. E. F——, of Atlanta, Ga., who was in labor with her twelfth child. Upon a vaginal examination, the mouth of the womb was found to be somewhat dilated, so that, under the influence of pain, the outline of the circular margin corresponded to the size of a silver dollar. The presentation being that of the vertex, with occiput to the front, and the soft parts in a state predisposed to yielding, with the vaginal secretion sufficient for lubrication, while the uterine contractions recurred at regular intervals, not exceeding five minutes, I felt assured of a speedy and favorable termination of the labor. Upon inquiry, I learned that the bowels had been moved under the influence of a mild laxative during the morning, and that the bladder had been evacuated within the previous half hour, so that there seemed to be no indication for interference. With the history of happy delivery on previous occasions, I felt satisfied to let well enough alone, and await developments in the full exercise of that masterly inactivity which is urged by Dr. Corson. Learning that quick time had been made in her former confinements, it was thought most prudent to await developments in the chamber of the parturient. The neglect of this precaution has sometimes involved the attendant in the very awkward predicament of being called to find the baby born, and produced the impression of neglect of duty upon the patient as well as those sympathizing friends who are generally at hand on such occasions.

So soon as the sound of rushing waters informed me that the membranes had been ruptured, I repaired at once to the bedside, laying off my coat and preparing for action ; but had only time to get the husband to unbutton and remove my cuffs before the head was protruded through the vulva, thus being committed to complete the delivery without rolling up my sleeves, which I consider as proper for this undertaking, in preference to soiling them, or failing to render efficient service for fear of such a result. It is not a matter of taste here which comes into play, in having the sleeves above the elbows for the due performance of this service ; and though the obstetrician assumes the air of a butcher in rolling up his sleeves to commence work, he avoids the revolting aspect of blood-stained sleeves when the job is finished, so that he is not under the necessity of borrowing a shirt before he can leave the house. It shows not only good sense, but a consideration of duty, in being ready to render any assistance during the delivery of the child and the expulsion of the afterbirth, or the subsequent management of hemorrhage, by passing the hand and arm within the parts, as is sometimes requisite.

Immediately upon the delivery of the child, it was ascertained that the umbilical cord was around its neck, and turning the head towards the vaginal outlet, to relax the tension, I removed a coil by passing the cord over the head, supposing that all the trouble was ended. But it was soon discovered, by the stifled cry of the child and discolored surface, that there was more or less asphyxia remaining, so that my fingers were again directed to the child's neck to find another coil still around it, which was quickly removed, as in the first instance. The desire to avoid unnecessary exposure of our female patients, under such circumstances, should not prevent such ocular examination as may be requisite for the safety of mother and child in cases of threatened strangulation or rupture of the cord, from being put on the stretch upon the delivery of the child. Although the sense of touch may convey clearly the impression of tension upon the umbilical portion of the cord before the buttocks are delivered, and we may feel the loop around the neck, it is too tight for removal when passing twice around the neck of the child. It will be perceived that the separation of the child to the usual distance from the mother, with such shortening of the connecting link as results from the cord being coiled twice around the neck, as in this instance, may create a strain upon the placental attachment so as to lacerate the fibers and thus expose the mother to risk of hemorrhage. The indications are met most efficiently by keeping the head of the child near the vulva, and proceeding with all possible dispatch to relieve the constriction by reversing the turns of cord over the head, or imparting a rotary movement to the child's body so as to uncoil the cord from its neck.

After waiting until the crying of the child indicated free respiration, and the superficial discoloration had yielded to the ordinary hue under the restoration of the circulation, observation satisfied me that all was right, so that the cord was tied in two places by a double loop of soft cotton thread, and cut between, leaving about two inches next the navel of the child. The importance of ligating so as to admit of placing another ligature on the cord was illustrated, by free bleeding afterwards, in this case, so that it was requisite to secure the cord by another ligature within twelve hours after the first was applied. In the face of such facts it is incomprehensible how ligation can be omitted. Having ascertained by manual examination over the walls of the abdomen that the womb was contracting upon the placenta, and giving the mother an assurance that her anticipation of twins would not be realized, my attention was directed to an inspection of the child, to verify that it was perfectly formed in all respects—which strikes me as obligatory upon every accouchier, and should not be omitted by the younger members of the profession who engage in obstetric practice. I likewise prepared four pieces of old linen, about four inches square, with holes cut through the center of each by the scissors, into which the cord was passed, and then laid upward in the axis of the body upon the upper layer of the cloth. Two of the adjoining layers were then folded from either side over the cord, and the lower limb of this fold was brought up and laid upon the navel. Over this the two remaining layers next to the abdominal surface were folded longitudinally, and the belly-band was passed over this and around the body of the child, to be secured by safety-pins in front. This belly-band is considered not only unnecessary but a hurtful restraint by some newlight obstetricians, yet the neglect of it most assuredly leaves the unsupported navel liable to undue protrusion.

After waiting nearly a half hour without the expulsion of the placenta, and observing that the recurring pains indicated uterine contraction, the left hand was placed over the abdomen, while the right was directed to the neck of the womb. An unusual shape of the fundus and body of the womb, with evident increase of size beyond what was noted in the previous examination, induced me to press the fingers of the right hand up over the posterior margin of the placenta, still entirely within the cavity of the womb, and apparently blocking the outlet. By the aid of gentle traction upon the cord, with the left hand, the edge of the placenta was hooked down by the fingers of the right, when the left hand was again carried over the womb with a gentle grasping to favor its contraction. Upon the expulsion of the placenta there was an immense gush of blood, which was relieved by the prompt contraction of the womb and the vigorous manipulation externally. But there was now observed more than before the delivery of the placenta, a lateral extension with a flattening of the womb, in the antero-posterior measurement, giving the outline of the Indian stone-axe, which presented an irregularity indicating a spasmodic contraction of the uterine fibers, such as I had never observed previously in any case of labor, throughout an extended experience in this department, and the observation of many irregular uterine contractions after delivery of the child. But as the hemorrhage might recur upon the relaxation of the abnormal state of the muscular tissues, I ordered the fluid extract of ergot to be obtained at once, so as to be in readiness if needed, and gave the patient strong brandy toddy immediately, with directions to repeat it occasionally. Considering the uniform support of the broad abdominal bandage as especially indicated by the existing irregular uterine contraction, it was applied with moderate firmness, and, in view of the facts noted, I thought it incumbent to remain with my patient and watch the progress of the case. In about an hour abdominal pains, with a restless anxiety, presented the usual indications of internal hemorrhage, which were confirmed upon a special examination over the womb and by the vaginal canal. The cervix uteri was clogged by a dense coagulum, which seemed to have an almost fibrinous firmness, and was evidently continuous with a mass within the uterine cavity, which distended the walls so as to produce a very perceptible enlargement of the body of the womb, under palpitation through the abdominal parities. The coagulum embraced by the neck of the womb had the dumb-bell formation, from the compression of the circular fibers around it, and this effectually prevented the further discharge of blood, although there was evidence of previous outward flow in the coagula within the vagina and outside the vulva. Detaching this plug from the neck of the womb and removing the clots from within its cavity, while gentle grasping movements were kept up with the left hand over the body of the organ, I was surprised to discover that, upon contraction, the outline of the womb was of the same irregular shape. A half teaspoonful of Wyeth's fluid extract of ergot, with a tablespoonful of brandy and a little water, was given immediately ; but as she subsequently became nauseated and vomited, the brandy was repeated without the ergot. There being no return of hemorrhage, and the womb assuming a more natural form, while still contracted, at the end of six hours I left my patient with instructions to take a cup of tea, and only to use the ergot in the event of appearance of the hemorrhage externally, or distension of the abdomen in the uterine region from internal hemorrhage.

