# Correction

**Published:** 1886-08-20

**Authors:** 


CORRECTION.—In referring to Dr. Gaston's contributions to the "Reference Hand-book," in our notice of that work last month, an error occurred in the titles, which should be corrected and read as follows: Cholecystectomy, Cholicystotomy, and Duodeno-Cholecystostomy.

